# Low-Cost Hyperspectral Imaging with A Smartphone

**DOI:** 10.3390/jimaging7080136

**Published:** 2021-08-05

**Authors:** Mary B. Stuart, Andrew J. S. McGonigle, Matthew Davies, Matthew J. Hobbs, Nicholas A. Boone, Leigh R. Stanger, Chengxi Zhu, Tom D. Pering, Jon R. Willmott

**Affiliations:** 1 Department of Electronic and Electrical Engineering, University of Sheffield, Sheffield S1 4DE, UK; mbstuart1@sheffield.ac.uk (M.B.S.); matt.davies@sheffield.ac.uk (M.D.); m.hobbs@sheffield.ac.uk (M.J.H.); nick.boone@sheffield.ac.uk (N.A.B.); leigh.r.stanger@gmail.com (L.R.S.); cz374@cam.ac.uk (C.Z.); 2 Department of Geography, University of Sheffield, Sheffield S10 2TN, UK; a.mcgonigle@sheffield.ac.uk (A.J.S.M.); t.pering@sheffield.ac.uk (T.D.P.); 3 Cambridge Advanced Imaging Centre, University of Cambridge, Cambridge CB2 3DY, UK

**Keywords:** hyperspectral, smartphone, low-cost, environmental monitoring, field deployable, portable

## Abstract

Recent advances in smartphone technologies have opened the door to the development of accessible, highly portable sensing tools capable of accurate and reliable data collection in a range of environmental settings. In this article, we introduce a low-cost smartphone-based hyperspectral imaging system that can convert a standard smartphone camera into a visible wavelength hyperspectral sensor for ca. £100. To the best of our knowledge, this represents the first smartphone capable of hyperspectral data collection without the need for extensive post processing. The Hyperspectral Smartphone’s abilities are tested in a variety of environmental applications and its capabilities directly compared to the laboratory-based analogue from our previous research, as well as the wider existing literature. The Hyperspectral Smartphone is capable of accurate, laboratory- and field-based hyperspectral data collection, demonstrating the significant promise of both this device and smartphone-based hyperspectral imaging as a whole.

## 1. Introduction

The rapid progression of smartphone technologies in recent years has led to a significant uptake in smartphone-based components in the design of optical sensing technologies. The inclusion of these consumer market components provides a significant advantage to device developers due to the substantial reduction in costs provided by the off-the-shelf nature of these components [1,2]. Furthermore, the increased processing power and the high-resolution camera systems often associated with these low-cost devices provides substantial opportunities to develop state-of-the-art optical sensing technologies at a fraction of the cost of currently available systems without compromising on the data quality captured. To date, smartphone technologies have been utilised in a variety of devices such as smartphone spectrometers [3,4,5] and multispectral sensors [6,7,8]. These devices have been implemented in a wide variety of settings, from point-of-care analysis to environmental monitoring applications. As such, smartphones represent one of the most promising platforms for the development of cost-effective portable measurement technologies.

More recently, there has been a drive toward developing smartphone-based hyperspectral imaging to provide a level of data resolution not possible with single pixel and multispectral designs. A number of studies have produced low-cost hyperspectral imagers either comprised of smartphone components or capable of communication with a smartphone for data viewing processes, e.g., [9,10,11,12]. Furthermore, studies such as those of He and Wang [13] and Park et al. [14] have developed smartphone-based hyperspectral imagers through the virtual transformation of the RGB data acquired by the built-in camera sensor. Whilst the examples above demonstrate that accessibility is beginning to improve, affordable, field deployable hyperspectral sensors remain in short supply across a wide range of application areas [15], with the increasing focus on our changing climate highlighting the growing need for low-cost, field deployable hyperspectral sensors across environmental monitoring applications. By developing a range of low-cost, portable hyperspectral sensors, we increase the potential for valuable data collection across a wider range of key environmental areas. This, in turn, allows us to improve our understanding of the processes that effect these highly important, dynamic environments.

To date, very low-cost spectrometers have been designed for incorporation with smartphone devices. These devices have largely been designed as teaching aids, providing easily accessible, educational set-ups, for example, Public Lab [16], presents a simple papercraft spectrometer, where a cardboard housing and a simple diffraction grating utilising a digital video disc (DVD) fragment are used to capture spectral datasets from a smartphone camera. Whilst this simple design does allow for spectral datasets to be observed, its basic components limit its potential uses, for example, the use of a DVD fragment as a diffraction grating substitute can introduce potential data quality issues, resulting from scattered light interference as well as transmittance shifts leading to the intensity variations of imaged objects at each wavelength of interest [17,18,19]. Despite these limitations, this basic design highlights the potential availability of low-cost, accessible, smartphone-based data collection.

In this article, we aim to demonstrate the opportunities available for low-cost smartphone-based sensing units through the construction of a low-cost unit capable of accurate, field-based data collection. Using the Public Lab spectrometer design as a foundation for further innovation, we introduce a low-cost, field deployable system where a standard smartphone can be utilised as a hyperspectral imager through the addition of a 3-D printed attachment. This device is capable of data capture across the visible spectrum (400–700 nm) and represents a valuable, easily implemented design, which, to the best of our knowledge, is the first fully incorporated smartphone-based hyperspectral imaging system of its kind. In this article, we will describe the design and build process required for this instrument. We then demonstrate its efficacy in a variety of environmental applications, providing a clear comparison to the existing literature and our previous work [1]. In so doing, we highlight the standard of data collection possible with such a low-cost, portable design.

## 2. Materials and Methods

### 2.1. The Hyperspectral Smartphone

**Figure 1:**
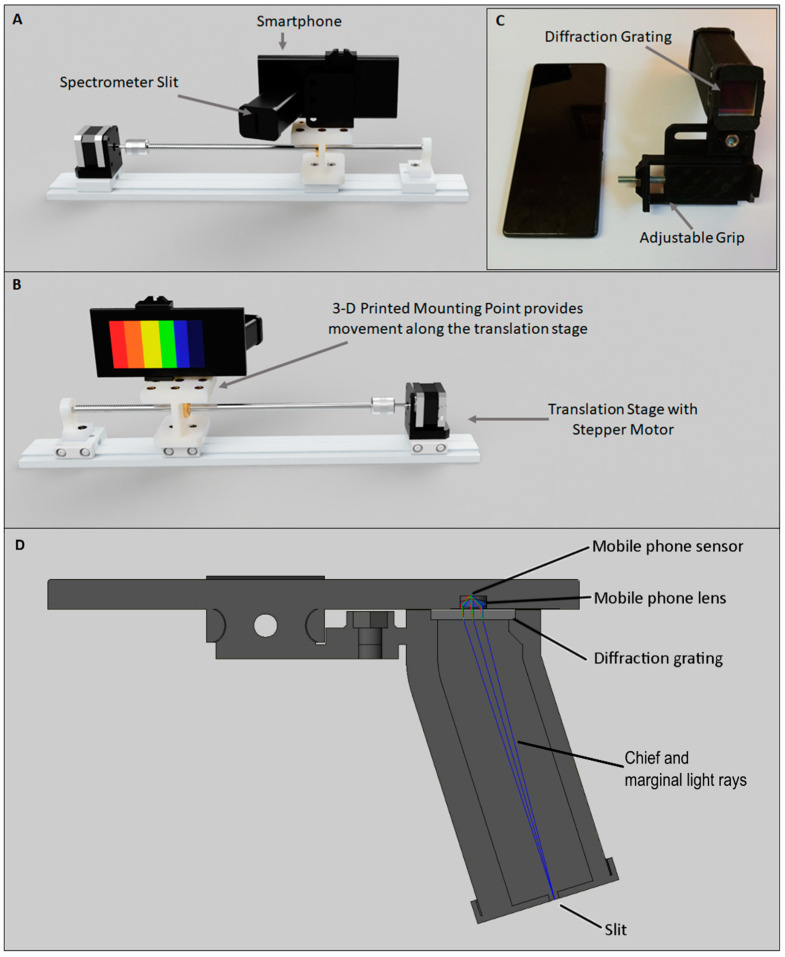
Schematic diagram of the Hyperspectral Smartphone mounted to the translation stage. (**A**) and (**B**) show the front and rear views, respectively, (**C**) shows the Hyperspectral Smartphone attachment prior to connection with a smartphone, highlighting the location of the spectral optics. (**D**) shows a cross section of the smartphone spectrometer system and shows how the marginal and chief rays travel through the system.

The Hyperspectral Smartphone (Figure 1) is comprised of an easily attachable 3-D printed spectral housing containing an Edmund Optics transmission diffraction grating (#49-580). This instrument is intended to be versatile, and as such the attachment grip and spectral housing position are adjustable, increasing usability across a variety of smartphone widths and camera configurations. The 3-D printed housing provides a robust structure, increasing the overall reliability of this design. Furthermore, a standard slit is present within the printed design, which can be narrowed to best fit the intended target scene. The diffraction grating is positioned directly in front of the smartphone camera sensor. To minimise potential light leaks at this location, cushioning foam has been attached to provide a secure fit between the smartphone and spectrometer components. This new design can be easily and reliably attached to a wide range of smartphone devices allowing for accurate, repeatable data collection in a range of environmental settings. Furthermore, the optics provide substantial improvements to the quality of data capture possible without introducing significant costs. The Hyperspectral Smartphone costs ca. £100, making it a highly accessible, low-cost hyperspectral instrument when compared to currently available portable hyperspectral imagers in commercial markets.

### 2.2. Image Capture and Data Processing

**Figure 2:**
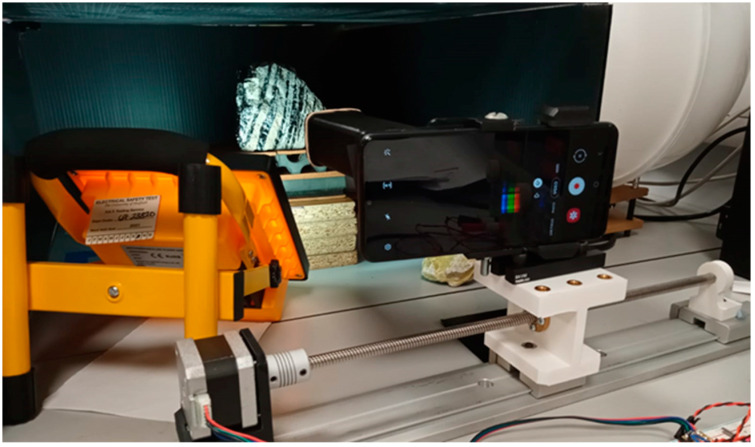
The Hyperspectral Smartphone mounted on the translation stage ready to image an obsidian flow banded ash tuff rock within a laboratory setting.

The Hyperspectral Smartphone is a push broom style sensor that can be used as either a hand-held or tripod-based instrument; however, it should be noted that, due to operator shake, datasets captured as a hand-held device can be subject to greater distortion. To minimise these effects, the device can be mounted on an automated translation stage, where a stepper motor is used to track across the target scene. The slit-width of the system is 0.5 mm and the slit-lens distance is 90 mm. The inclusion of the translation stage allows for stable, repeatable scene passes, resulting in clearer datasets. Whilst it remains possible to collect datasets by hand, the use of the translation stage results in much clearer imagery. Data collection was completed using the video function of the smartphone camera. For all data collection, the video frame rate was set at 30 fps. Once recording, the device was tracked, left to right, across a scene at 1 mm/s intervals across the target. When recording scenes within a laboratory setting, the target object is placed within a dark box to minimise the interference of ambient stray light and is illuminated using a 20 Watt LED lamp with a diffuser to minimise bright spots within the scene. Figure 2 shows the Hyperspectral Smartphone ready for data collection within a laboratory environment. As mentioned above, the instrument is versatile and can, therefore, be used with a wide range of smartphones, however, for the purposes of this article, the smartphone used was a Samsung Galaxy A12. The presence of an auto-focus feature within the smartphone camera software is of significant benefit to this instrument, minimising the specialist knowledge required to operate the Hyperspectral Smartphone, and as such, providing a versatile, accessible, user-friendly system. A full list of camera specifications for the Galaxy A12 can be found within Table 1. The instantaneous field of view (IFOV) [20] for each pixel within the spectrometer image was measured to be approximately 5 mm × 5 mm for a 95% energy enclosure at a working distance of 300 mm. Whilst the total field of view (TFOV) is determined by several factors. At short working distances (on the order of the travel distance) the vertical field of view (VFOV) is determined by the slit height and the distance between the slit and the grating, and the horizontal field of view (HFOV) is determined by the travel distance of the phone. At greater working distances, the acceptance angle of the slit (which is determined by the slit width, height, and distance to the grating) makes a greater contribution to both the HFOV and the VFOV. No additional coupling optics were incorporated before the spectrometer slit.

Once capture was complete, individual frames were extracted from the video file. Dark and white reference frames were also captured at this stage. To obtain a white reference a piece of matt white card was positioned in place of the target object and illuminated in the same manner. To correct the image frames for illumination and sensor biases whilst preserving the colour data of the output, the following analysis was implemented in Matlab for each colour region within each frame.

(1) $$target_{\lambda }=\frac{\left(target_{raw\;\lambda }-dark_{\lambda }\right)}{\left(white_{\lambda }-dark_{\lambda }\right)}\cdot \frac{\left(white_{\lambda_{1}}-dark_{\lambda_{1}}\right)}{\left(target_{raw\;\lambda_{1}}-dark_{\lambda_{1}}\right)}$$

where *target* represents the object to be imaged, and *dark* and *white* represent the dark and white references, respectively. The subscript *raw λ* represents an uncorrected spectrum.

In order to calibrate the instrument and to enable samples from different sources to be accurately compared, a radiometric assessment of the optical power represented by each pixel within the image was performed by measuring the power reflected by the white reference target. This was performed using a photodiode-based radiometer, as described by Zhu et al. [21], but with its RG850 long-pass filter replaced by a narrow bandpass filter centred on 550 nm with a full width half maximum of 10 nm (Thorlabs Stock #FB550-10, Ely, UK). By comparing the photocurrent measured by the radiometer with and without the filter in place, the reflected optical power collected by the radiometer could be calculated. Given that the FOV of the radiometer represented an area upon the target of approximately 14 mm in diameter at its 1 m operating distance, the reflected power per unit area, without the filter in place, was calculated from this to be approximately 46.55 uW/m^2^. Therefore, the optical power reflected from the white reference target was estimated to be 7.17 nW. For a comparison, the radiometer was sighted at the LED lamp directly, resulting in an optical power measurement of 108.62 nW. These values show that the optical throughput (related to etendue [22]), is approximately 6.6%.

Given that each pixel within the image of the spectrometer represents an area of 5 mm × 5 mm, the power collected in total per pixel of the spectrometer is approximately 1.16 nW for the white reference target. In the author’s experience, a typical “rule of thumb” for the development of optical instrumentation is that a good signal to noise ratio can be achieved using a silicon-based detector measuring 1 nW in 1 μm.

In order to calibrate the instrument spectrally, and to extract a spectral response curve from each corrected image frame, we utilised the calibration process within the Spectral Workbench software. This software is also publicly available and accessible from Public Lab [23], further demonstrating the accessible nature of this instrument. To complete the calibration accurately, a separate calibration image needed to be captured of a fluorescent lamp. Compact fluorescent lamps contain mercury vapour, which, when energised, emits a consistent, characteristic spectrum. Accurate wavelength ranges can then be applied to the image frames by aligning the peaks present at 436 nm and 546 nm within the software with their respective peaks in the calibration image. The resulting spectral curves were then averaged to provide one comparable spectrum for each target, allowing for direct comparisons to be made between different days and/or different targets. In its current format, the presence of a Bayer colour filter within the smartphone optics will reduce the sensitivity of the measurements captured with this instrument, however, as demonstrated below, we have been able to produce accurate, high quality, qualitative datasets, and as such we highlight the substantial potential available within this area of research.

Finally, spatial datasets were created in Origin Pro (2020b) by extracting the pixel values from a specific wavelength/column within each image frame from the chosen scene. These columns were then combined to create a spatial dataset of the target object that displayed the spectral response captured from a specific wavelength.

## 3. Results and Discussion

### 3.1. Applications

The laboratory-based environmental applications discussed in Section 3.1.1 and Section 3.1.2 replicate the experimental work conducted in Stuart et al. [1]. We decided to replicate these experiments as they can be simply implemented and produce rapid results that easily demonstrate the capabilities of the hyperspectral sensor when the resulting datasets are compared to the existing literature. Furthermore, accurate, low-cost hyperspectral alternatives remain somewhat absent in these contexts at present, therefore, we aim to provide a foundation for the development of low-cost hyperspectral data collection in these application areas. As such, a full explanation of these experiments and their importance to their relevant fields can be found in Stuart et al. [1]. We then go on to compare the Hyperspectral Smartphone to a pre-existing, laboratory-based hyperspectral imager in Section 3.2 before highlighting the capabilities of the Hyperspectral Smartphone as a field-portable device in Section 3.3, where the instrument is utilised in a field setting for the collection of both vegetation and wider landscape datasets at different working distances.

#### 3.1.1. Fruit Quality Control

**Figure 3:**
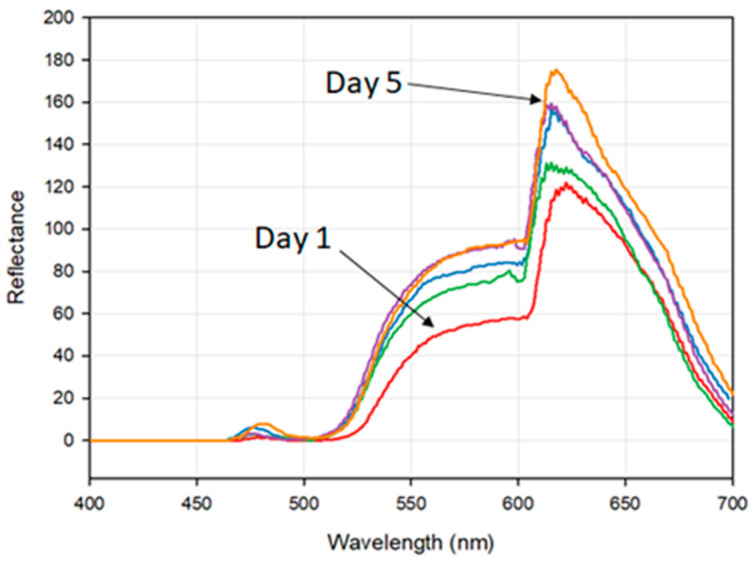
The observed spectral response curves of a healthy apple over the five-day measurement period. Note the general increase in reflectance over the measurement period and the absorption features present at ca. 550 nm and ca. 650 nm.

Hyperspectral images of a healthy apple were captured every 24 h over the course of five days in order to observe any potential changes in spectral response associated with the breakdown of pigments during the fruit ripening process [1,24,25]. Figure 3 shows the observed spectral response curve of the fruit over the five-day measurement period. A clear increase in reflectance over time can be observed, which is to be expected during the ripening process [26,27], emphasising the Hyperspectral Smartphone’s capability to detect accurate spectral data. Furthermore, absorption features associated with the pigmentation of the fruit are evident within the dataset; most notably a ‘shoulder’ starting at ca. 550 nm associated with anthocyanin absorption and a slight ‘shoulder’ at ca. 650 nm highlighting the absorption of chlorophyll b [24,28]. The literature also highlights an absorption feature for chlorophyll a, which is represented by a distinct loss in reflectance at ca. 675 nm followed by a rapid increase in reflectance towards the infrared [24,26,27,28]. Whilst a loss in reflectance is observed in our dataset in this region, the subsequent increase is not present. This is believed to be due to the spectral range of the Hyperspectral Smartphone, as this absorption feature is present close to the upper boundary of the instrument’s spectral range. We therefore infer that the reduced sensitivity associated with the edges of the spectral range of the diffraction grating may be resulting in some data loss in this region. Despite these losses, it is clear that the Hyperspectral Smartphone is capable of capturing accurate spectral datasets that are comparable with the existing literature in this field.

**Figure 4:**
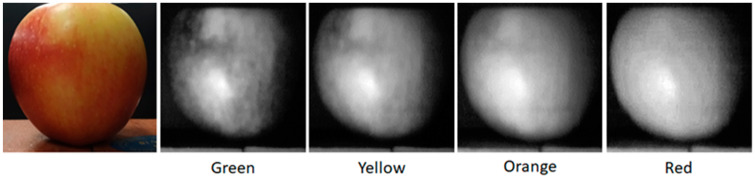
Spatial datasets of the apple across different wavelengths. Note the variations in pigments across the spectrum.

The abilities of the Hyperspectral Smartphone are further illustrated in Figure 4, which demonstrates clear spatial data collection that clearly highlights the variations in pigmentation across the apple’s surface and correlates well with the observed spectral response curve. The clarity of the datasets obtained using the automated translation stage highlights the significant potential of this device, and indeed smartphone-based hyperspectral devices as a whole.

#### 3.1.2. Volcanic Rock Mineralogy

**Figure 5:**
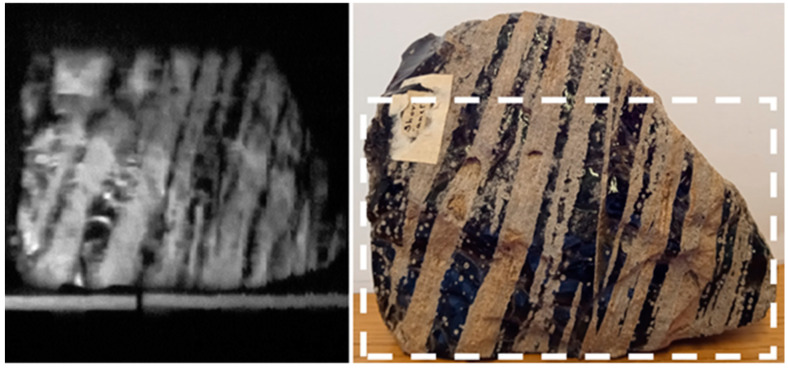
Spatial dataset obtained of an obsidian flow banded ash tuff, clearly highlighting the individual flow bands. Hyperspectral dataset taken from ca. 600 nm.

Volcanic rock images were also captured with the Hyperspectral Smartphone. These images were acquired as a means of demonstrating the device’s ability to identify variations and feature changes across a target object’s surface. Figure 5 shows the hyperspectral data captured of an obsidian flow banded ash tuff. As Figure 5 shows, this target has clear variations present across its surface. These variations are clearly replicated in the hyperspectral data, adding further support to the capabilities of this low-cost design. The banding across the rock is easily recognisable within the hyperspectral image, allowing for the straightforward identification of the individual flow bands.

**Figure 6:**
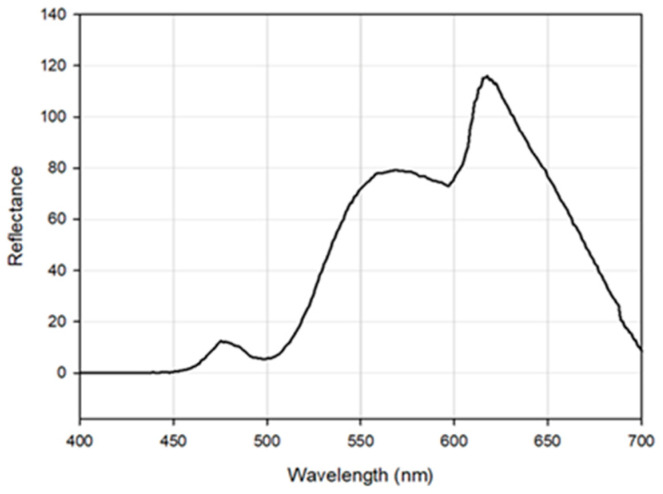
Observed spectral curve for a sulphur target. Note the clear increase in reflectance from ca. 500 nm.

**Figure 7:**
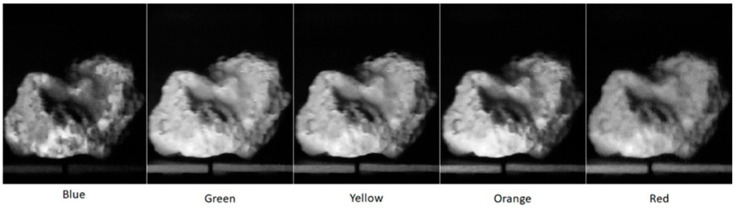
Spatial datasets for the Sulphur target highlighting the variations in reflectance across the different wavelengths.

To further demonstrate the spectral capabilities of this instrument, a sulphur rock was imaged. Sulphur was chosen due to its distinctive spectral response where a distinct increase in reflectance is evident from ca. 500 nm [29]. Figure 6 shows the spectral response of Sulphur collected by the Hyperspectral Smartphone. This figure clearly shows the expected increase in reflectance from ca. 500 nm, emphasising the capabilities of this instrument. The additional variations across the spectrum are likely to be the result of variations present across the surface of the sulphur target used due to the presence of colour variations across the rock’s surface, as highlighted in Figure 7. However, the key reflectance feature at ca. 500 nm remains prominent within the captured dataset.

### 3.2. Comparisons to A Pre-Existing Low-Cost Imager

To examine the capabilities of the Hyperspectral Smartphone in more detail, we compared them directly to the low-cost hyperspectral device presented in Stuart et al. [1]. Table 2 provides a direct comparison between the Hyperspectral Smartphone and the laboratory-based hyperspectral imager. As Table 2 highlights, both instruments represent valuable, low-cost hyperspectral sensors that can be deployed with relative ease and without the need for extensive additional set-up time. Both devices are capable of a range of image capture scenarios and allow the operator to vary the image dimensions to fit the target scene, either through manually editing the dimensions prior to scene capture (Laboratory-based Hyperspectral Imager) or through the extension of scene capture sweeps (Hyperspectral Smartphone). Whilst the Hyperspectral Smartphone is constrained to the settings available within the built-in smartphone software, it represents a significantly cheaper and therefore more accessible, hyperspectral imaging sensor, when compared to the laboratory-based imager. Furthermore, the presence of an auto-focus feature within the smartphone software allows the Hyperspectral Smartphone to be especially user friendly. However, its spectral range is limited to the visible spectrum, whereas the laboratory-based imager is capable of a broader spectral range and can be converted to cover different regions of the spectrum, such as infrared, with relative ease [1]. The prominent differences present between both instruments are largely a result of the cost of the components involved and their intended research area. The laboratory-based hyperspectral imager represents a more specialised instrument in this comparison because it is typically suited to bench-top laboratory analysis, whereas the Hyperspectral Smartphone represents a more accessible design that can be implemented in a range of settings outside the laboratory. As such, these differences are clearly represented in the set-up costs associated with each device.

**Figure 8:**
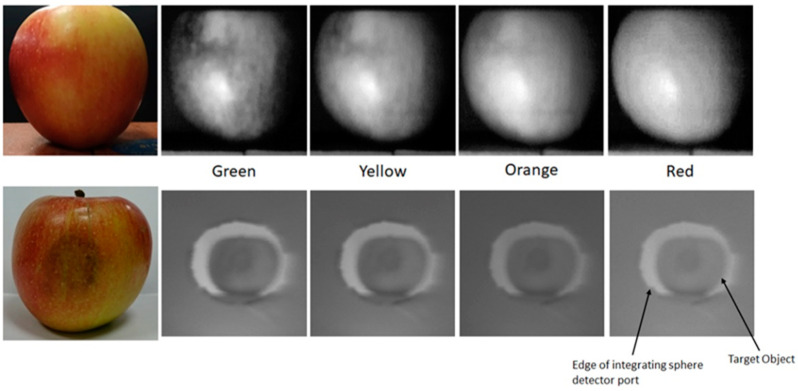
Side-by-side comparison of apple targets captured across different wavelengths by both the Hyperspectral Smartphone (top dataset) and the laboratory-based hyperspectral imager (bottom dataset). Note, that the apple target is placed within a low-cost integrating sphere during data capture with the laboratory-based instrument.

Figure 8 provides a side-by-side comparison of apple targets captured by both instruments. As this figure demonstrates, both the Hyperspectral Smartphone and the laboratory-based hyperspectral imager are capable of detecting pigment variations across this type of target. When comparing the two spatial datasets, the Hyperspectral Smartphone appears to be capable of greater image resolution, however, it should be noted that the optical system present within the laboratory-based instrument represents a basic set-up [1], and is, therefore, capable of improved image clarity, subject to the inclusion of an upgraded lens system. The Hyperspectral Smartphone benefits from the built-in optical system present within the chosen smartphone. As most smartphones are now capable of high quality image capture, it makes sense that the Hyperspectral Smartphone would be superior in the context of this comparison.

### 3.3. A Field-Portable Low-Cost Hyperspectral Imager

**Figure 9:**
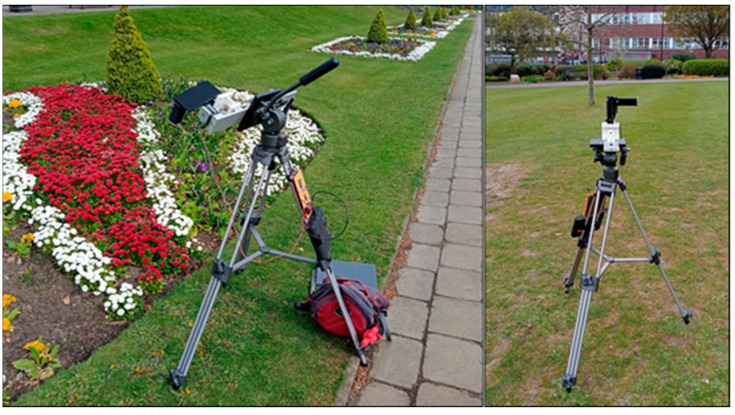
The Hyperspectral Smartphone ready for data collection within a field setting.

The above sections have demonstrated the Hyperspectral Smartphone to be a valuable low-cost instrument capable of high quality, accurate hyperspectral data collection within a laboratory setting. We now aimed to further test its capabilities as a robust, field-portable instrument. To do this, the Hyperspectral Smartphone, with the translation stage, was mounted onto a tripod to allow for stable data capture and the translation stage was converted to battery power. Figure 9 shows the Hyperspectral Smartphone in the field ready for data collection.

**Figure 10:**
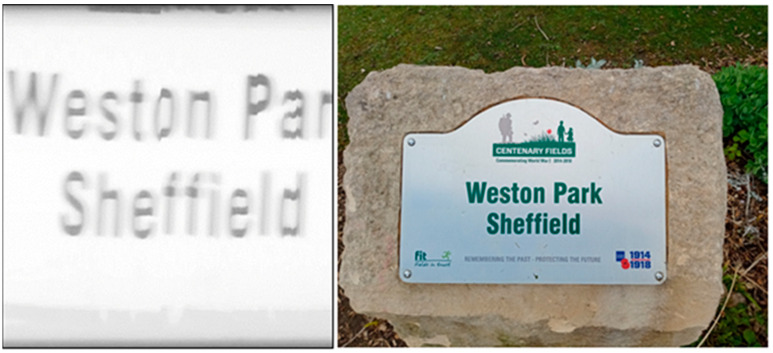
Spatial dataset captured of a sign within Weston Park over a short working distance. Note the clarity of the writing.

In the field, a variety of targets were imaged in order to determine the Hyperspectral Smartphone’s abilities in a field setting at a range of different working distances. For this data collection, measurements were completed within Weston Park, Sheffield. This site was chosen due to the wide range of potential targets available without the need for extended travel, allowing for initial field tests to be completed under COVID-19 travel restrictions. Datasets were captured at both short ca. 1 m and longer ca. 20 m working distances. Figure 10 shows the results obtained over a short working distance for a distinct target, in this case a sign within the park. It is clear from this figure that the Hyperspectral Smartphone is capable of clearly defining target object features, in this instance replicating the writing clearly within the spatial data.

**Figure 11:**
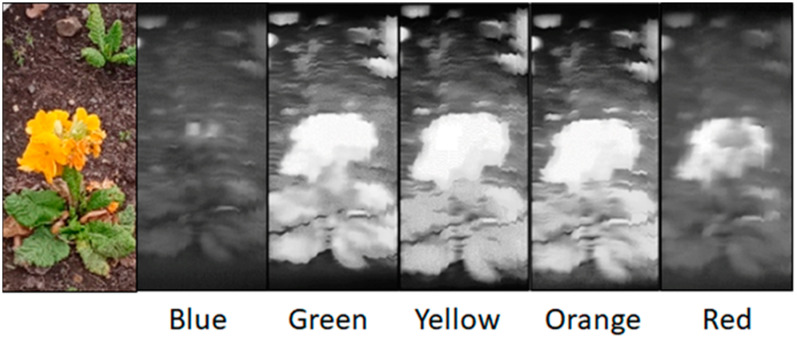
Spatial datasets across different wavelengths for a section of flower bed acquired over a short working distance.

More intricate targets were also imaged at this working distance. Figure 11 shows the spatial and spectral data collected from a section of a flower bed. Whilst some of the details of the target are lost within the spatial datasets, the spectral response variations remain clear following the expected response from this particular target. These datasets show the Hyperspectral Smartphone to be a valuable short-range field instrument.

**Figure 12:**
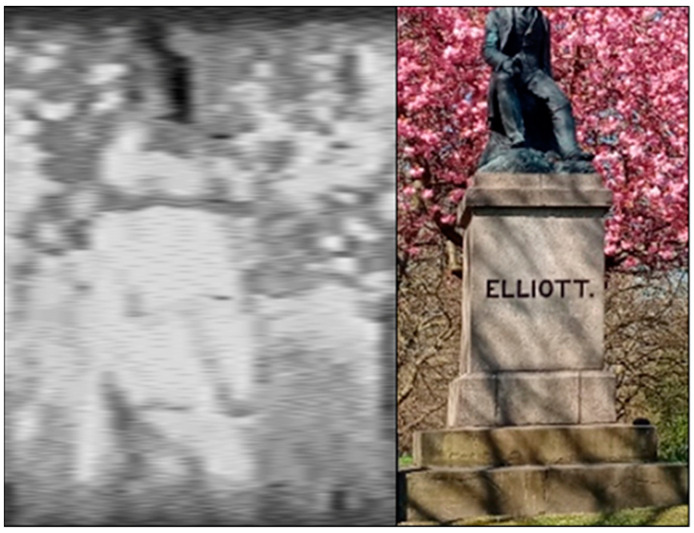
Spatial dataset captured of a statue over a longer working distance (ca. 20 m). Whilst this dataset is of poorer quality than others captured with this instrument, the target object can be identified highlighting the potential for the capture of landscape-style scenes in the future.

Finally, the Hyperspectral Smartphone was used to capture landscape-style datasets over a longer working distance. Figure 12 shows a spatial dataset captured over this range. Whilst this dataset is poor in comparison to the other data provided within this article, it is evident that there remains potential for the acquisition of these landscape-style datasets with this style of device. From these datasets it is clear that, in its current format, the Hyperspectral Smartphone is more suited to shorter range data capture, however, the preliminary data captured over longer distances shows promise, suggesting that with some minor modifications to the existing instrument landscape scenes may prove possible to accurately capture with this style of low-cost hyperspectral system.

## 4. Conclusions

In this article, we have documented, to the best of our knowledge, the first smartphone capable of hyperspectral data collection without the need for extensive post processing. We have discussed the design and build process required to convert a standard smartphone into a visible spectrum hyperspectral imager for ca. £100. Its abilities have been tested in a range of imaging scenarios and we have provided a robust metrology as well as a thorough comparison against the existing literature and a pre-existing low-cost hyperspectral imager. The spectral bandwidth and spectral resolution of the smartphone imaging system is comparable to that of the other low-cost imager. Both have variable imaging resolutions afforded by the scanning nature of the systems, and although the smartphone system has a finite vertical image resolution due to the number of pixels on the sensor, all these pixels scan the scene simultaneously, significantly decreasing imaging time in comparison to the lab setup. Portability is paramount in agricultural and environmental monitoring applications. It is clear that the Hyperspectral Smartphone is capable of accurate spectral and spatial data collection and has the potential for future deployment within a range of environmental monitoring applications, with the additional benefits of its portability and user-friendly setup, making it a valuable instrument for more remote field campaigns. The results obtained by the Hyperspectral Smartphone demonstrate the significant potential available in the field of smartphone-based hyperspectral imaging and, as such, represents a valuable addition to currently available field-portable hyperspectral technologies, providing a solid foundation for future development and improvements in this area of research.

## Figures and Tables

**Table 1:** Camera specifications for the Samsung Galaxy A12 smartphone.

	Samsung Galaxy A12
Resolution	48 MP (1920 × 1080 for video)
F Number	2.0
Focal Length (mm)	26
Fps	30

**Table 2:** Direct comparison between the Hyperspectral Smartphone and the laboratory-based hyperspectral imager.

	Hyperspectral Smartphone	Laboratory-Based Hyperspectral Imager
Imaging Mode	Push Broom	Whiskbroom
Approximate Cost of Instrument ^1^	~£100	<£6000
Image Capture Dimensions	Variable—can be modified by the operator.	Variable—can be modified by the operator.
Spectral Range (nm)	400–700	340–850
Spectral Resolution (FWHM nm)	14	12
Operator Input Options	Limited. Exposure settings can only be modified within the constraints of the built-in smartphone software.	Variable. Exposure settings and image parameters can be modified by the operator.
Potential Portability	Highly portable. Can be deployed anywhere with sufficient lighting. Capable of both indoor and outdoor data capture.	Limited portability within a laboratory setting.
Additional Equipment Required for Successful Data Capture	Additional illumination required for indoor data collection, e.g., an LED lamp. A fluorescent light is required to complete the data calibration process.	Additional LED illumination required. Target object placed in a low-cost integrating sphere during data capture.

^1^ These values represent the cost associated with the spectral set-ups and do not include potential additions such as LED lamps. However, these additions are often commonplace within laboratory environments or can be easily purchased for minimal additional cost.

## Data Availability

All relevant data are shown in the paper or could be recreated by following the methodology in the paper.
